# The Current State of Vascular Surgery Presence in Bilibili Video Platform of China

**DOI:** 10.3389/fsurg.2022.874113

**Published:** 2022-04-28

**Authors:** Haoliang Wu, Zhiwei Wang, Mingxing Li, Peng Sun, Liwei Zhang, Cong Zhang, Shunbo Wei, Boao Xie, Chunyang Lou, Zhentao Qiao, Yuanfeng Liu, Tao Bai, Hualong Bai

**Affiliations:** ^1^ Department of Vascular and Endovascular Surgery, First Affiliated Hospital of Zhengzhou University, Henan, China; ^2^ Key Vascular Physiology and Applied Research Laboratory of Zhengzhou City, Henan, China

**Keywords:** vascular surgery, video platform, media, Bilibili, vascular

## Abstract

**Background:**

With the development of the Internet, more and more patients search for disease-related information on video platforms during the treatment process, and physicians also look for learning materials through these video platforms. Bilibili is one of the most popular video platforms in China. This study evaluated information on various interesting topics, and related surgical procedures searched through Bilibili.

**Method:**

The Bilibili platform was independently queried for 12 common vascular diseases or related surgical procedures between October and November 2021 by two independent authors using the Baidu search engine. Information about the video and uploader was collected, and descriptive analyses of the overall and first-page results were performed.

**Results:**

A total of 3,998 search results were retrieved by searching 12 vascular-related topics, of which 2,225 actual videos (55.7%) were finally confirmed to be related to medicine. Videos for the public accounted for 84.8% of these 2,225 videos. In addition, 50.5% of the video results were uploaded by vascular surgeons, 12.4% by other specialties, 17.7% by organizations, and 19.4% by other individuals. The total number of videos searched for varicose vein and peripheral vascular diseases was the largest, and the total number of leg amputation videos was the smallest. The largest number of videos for medical professionals was about pulmonary embolism, and the smallest was about leg amputation. On the first pages, 168 results (70.0%) were actually medically relevant, and only 7.7% of the videos were uploaded by vascular surgeons.

**Conclusion:**

On the Bilibili platform, videos about vascular diseases are extensive but not comprehensive. The videos uploaded by vascular surgeons are rare, and the results searched are not precise. The online presence of vascular surgeons needs to be improved, which may partially solve the problem of low-quality videos due to the lack of strict management and censorship.

## Introduction

Traditionally, medical education requires a combination of clinical practice with textbooks, on-campus courses, laboratories, and lectures, and surgical education requires the accumulation of hands-on operative experience. Nowadays, Internet not only provides new methods for medical education but also provides new tools for patients and medical information providers. However, this is both an opportunity and a challenge, as the content of various video-sharing platforms has not undergone peer review and is not subject to strict regulations and censorship (1). Therefore, videos uploaded and evaluated by professionals are required.

Even with these drawbacks, there is no doubt that the video-sharing platform has become one of the important ways for people to obtain health information, and many new videos about diseases are uploaded every day (2). It is undeniable that these videos will provide people with a more convenient way and will answer some of their questions. One study showed that surgical trainees and practicing surgeons tend to use online videos as the primary resource for preparing surgical cases (3); this survey also found that most attending surgeons use online videos to prepare surgical cases (3). However, many recent studies on YouTube showed that not all videos about vascular surgery on the platform are beneficial. Research results on the video platforms on major lower limb amputations (4), lower limb arterial disease (5), carotid endarterectomy (6), abdominal aortic aneurysms (7), and common femoral artery access (8, 9) all showed that the current video platforms lack high-quality medical research videos uploaded by vascular surgeons. Unfortunately, this problem also appears in other fields of surgery (10–12). At present, most patients or their family members refer to information on the Internet during the onset or treatment process, so the lack of information from professionals is detrimental to them. Inaccurate videos uploaded by nonprofessionals can mislead patients and make it difficult to establish a trusting patient–physician relationship (13).

Recently, a study explored the current state of vascular surgery on Google and YouTube (2). Bilibili is a video platform integrating entertainment and education in China and one of the ways for patients and physicians to understand and learn health-related knowledge. The purpose of this research is to understand the overall situation of videos of several common diseases in vascular surgery and video uploaders on the Bilibili platform.

## Methods

**Figure 1:**
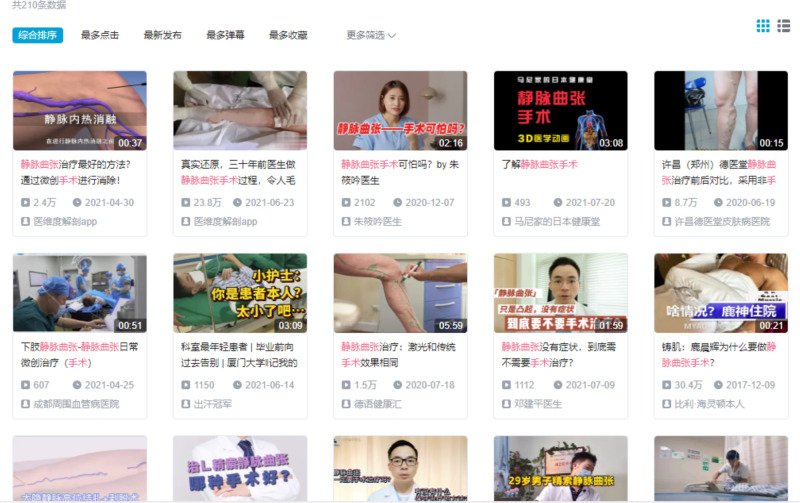
Sample result page on Bilibili.

A total of 12 common vascular diseases or related procedures were searched on the Bilibili media sharing webpage (http://www.bilibili.com) (Figure 1), which include aortic dissection (AD), endovascular aortic repair (EVAR), abdominal aortic aneurysm (AAA), deep vein thrombosis (DVT), carotid artery stenting (CAS), pulmonary embolism (PE), peripheral arterial disease (PAD), peripheral vascular disease (PVD), claudication, varicose veins, varicose vein surgery, and leg amputation. The search on the Bilibili media sharing webpage was conducted between October and November 2021 by two authors (H.W. and Z.W.) using the Baidu search engine. We collected all the video results and separately recorded the data of 20 videos on the first page of each keyword.

We first excluded the videos that were not related to medical science and then analyzed the remaining videos. These videos about medical science are defined as actual videos. The number of results retrieved directly is defined as the total number, and the number of actual videos is defined as the actual number. Then, we evaluated each actual video to determine whether the information was popular science or professional knowledge. Video information containing details of disease diagnosis, procedures, and management is considered professional knowledge. The search results containing recommendations from specialties and hospitals, personal stories of patients, disease introduction, disease risk factors, and simple introduction of treatment methods were considered popular science. Furthermore, each individual result was reviewed to record the uploader. Uploaders are divided into four categories: vascular surgeons, other specialties, organizations, and other individuals. The uploaders of the organization category included people from websites, applications, and WeChat public accounts. Finally, we calculated the proportion of videos containing professional knowledge and the proportion of uploaders in each category in the actual videos. The proportion of each topic category was based on the actual video number.

Given the use of publicly available data, institutional review board review and informed consent requirements were waived. Descriptive statistics were used to evaluate the results of the research. All analyses were performed using Microsoft Excel.

## Results

A total of 3,998 search results were retrieved by searching 12 vascular-related topics, of which 2,225 videos were finally confirmed to be related to medicine. Of these 2,225 actual videos, 84.8% were directed to the public. In addition, 50.5% of the video results were uploaded by vascular surgeons, 12.4% by other specialties, 17.7% by organizations, and 19.4% by other individuals. For 4 (AD, CAS, PE, and claudication) of the 12 search topics, vascular surgeons were mentioned less frequently than the other medical/surgical specialties.

**Figure 2:**
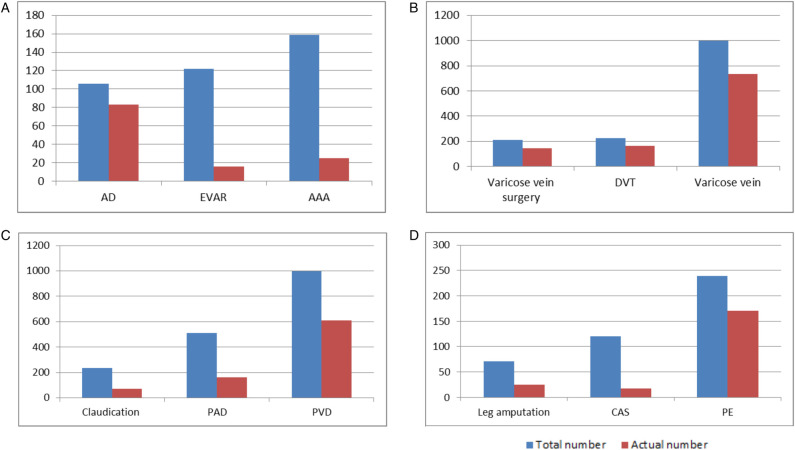
The total number of results and the number of actual videos. (**A**) Aortic results, (**B**) venous results, (**C**) peripheral arterial results, and (**D**) miscellaneous results.

The total number of videos searched for varicose veins and PVD was the largest, and the total number of results searched for leg amputation was the smallest. The number of actual videos searched for varicose veins and PVD was the largest, and the actual number of results searched for EVAR and CAS was the smallest. Nevertheless, the highest proportion of actual videos appeared when searching AD (78.3%), and the lowest proportion appeared when searching EVAR (13.1%) (Figure 2). The most-watched actual video was about DVT and PE (1.374 million), and the least-watched actual video was about EVAR (2,919).

**Figure 3:**
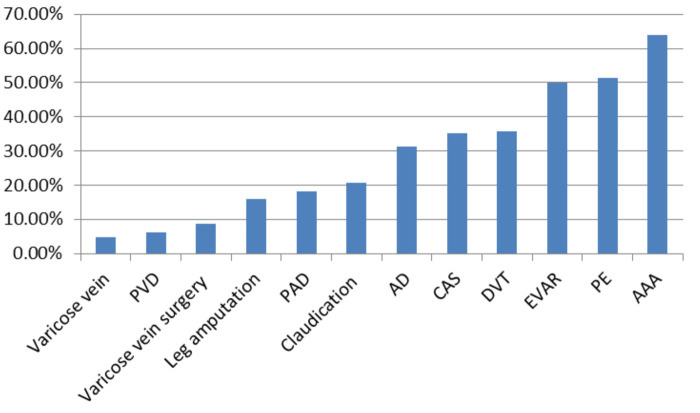
The proportion of video results for medical professionals.

**Figure 4:**
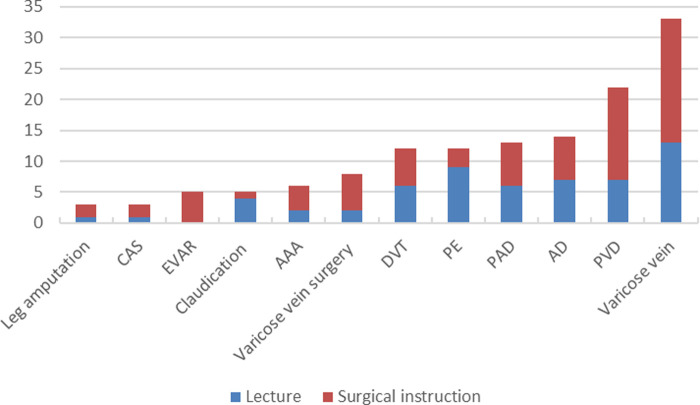
The number of videos about lectures and surgical instructions.

Most videos for medical professionals were about PE, and the least videos were about leg amputation. However, the highest proportion of videos for medical professionals was about AAA (64.0%), and the lowest was about varicose veins (4.8%) (Figure 3). Results of the lectures and the surgical instructions were also compared (Figure 4).

**Figure 5:**
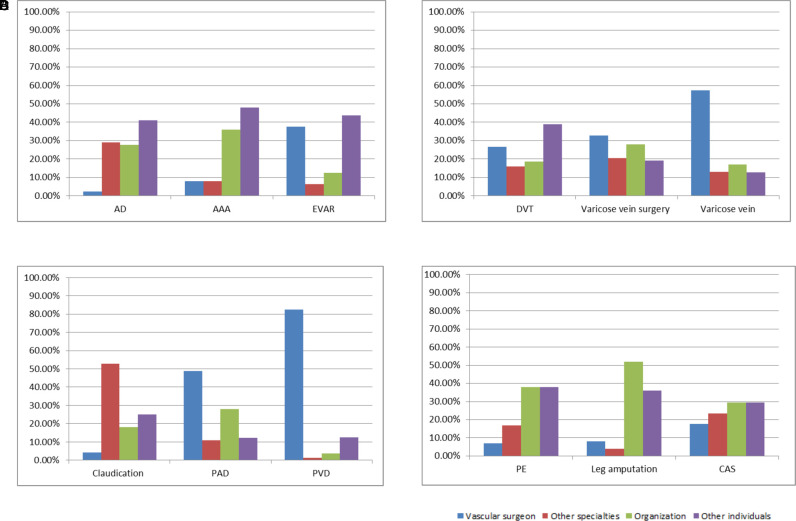
The proportion of videos uploaders. (**A**) Aortic results, (**B**) venous results, (**C**) peripheral arterial results, and (**D**) miscellaneous results.

The least results uploaded by the vascular surgeons were about AD, AAA, CAS, claudication, and leg amputation; the lowest percentage was about AD, PE, AAA, claudication, and leg amputation; and the largest number and proportion of videos uploaded by vascular surgeons were about PVD (Figure 5).

### Results on the First Page

**Figure 6:**
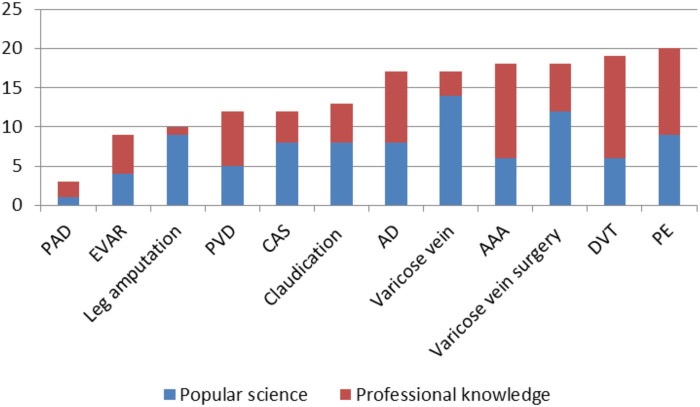
The number of popular science and profession-related results on the first pages.

Of the 240 results, it was finally determined that 168 results (70.0%) were actually medically relevant, of which 46.4% (78/168) of the videos were directed to the medical professionals (Figure 6). The videos uploaded by the vascular surgeons were present in the first 20 results for 50.0% of the search topics (AAA, EVAR, DVT, CAS, leg amputation, and varicose vein surgery). In addition, 7.7% of the video results were uploaded by vascular surgeons, 22.0% by other specialties, 27.4% by organizations, and 42.9% by other individuals.

**Figure 7:**
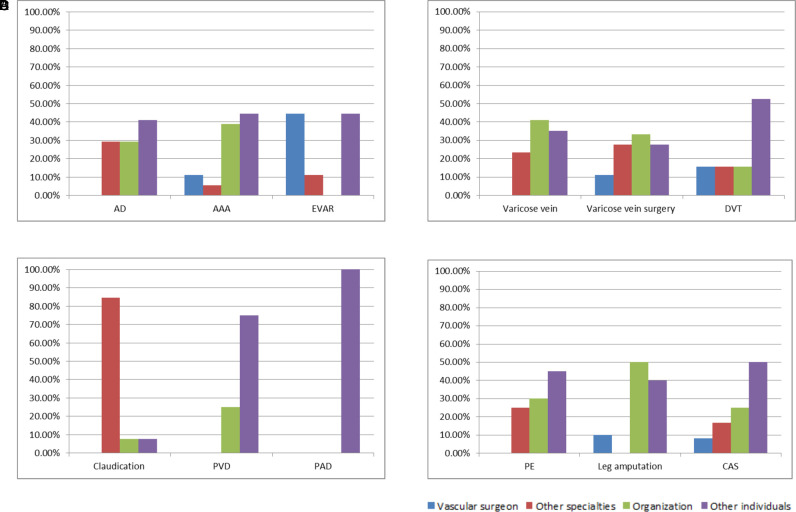
The proportion of video uploaders on the first pages. (**A**) Aortic results, (**B**) venous results, (**C**) peripheral arterial results, and (**D**) miscellaneous results.

Most videos for medical professionals were about DVT, and the least videos were about leg amputation. The highest ratio was about DVT (68.4%), and the lowest ratio was about leg amputation (10.0%). In the results on the first pages, the videos uploaded by the vascular surgeons did not present topics of AD, PE, PVD, PAD, claudication, and varicose veins. The largest number of results and the largest proportion (44.4%) uploaded by the vascular surgeons were related to EVAR (Figure 7).

## Discussion

In general, the number of results obtained by a direct search for each keyword was different (71–1,000), and the actual videos after screening did not account for a high proportion (55.7%) of the total. Only 15.2% of the videos were directed to the medical professionals, but the proportion in the first pages was three times that of the whole. The lack of videos uploaded by vascular surgeons results in insufficient technical quality, which may convey major biases to those who use these videos for training or even for laypeople to understand these diseases.

The Internet has become an important way for patients to obtain disease and treatment-related information because it can be accessed conveniently and always free of charge. When evaluating vascular surgery information on the Internet in 2012, it was found that most of the information was of low quality (14).

One study showed that average Internet users rarely clicked on results beyond the first page (2). Our results confirmed the importance of the first-page results after retrieval. Unfortunately, according to our results, the proportion of vascular surgeon uploaders on the first pages was much less than the overall average. Because there is still a significant knowledge gap in the experience and practice of vascular diseases between practicing physicians and vascular surgeons (15), the vascular surgeons should actively participate in creating popular science and profession-related videos, which can effectively improve the quality of videos.

In addition, the ranking of videos was also a problem, and researchers have raised the deficiency of a video ranking method of video platforms (11). The popularity of videos on YouTube is not calculated based on the quality of the video but based on a special algorithm, which may be view count and comments (11). One research about YouTube videos pointed out that although this may be a reasonable overall ranking method for online videos, it may be detrimental to education because of the quality; since accuracy is more important than popularity in education (11). Videos of Bilibili are also not sorted according to the quality of the videos but are sorted comprehensively based on similar algorithms. Although it is possible to perform new sorts based on the number of video clicks, upload time, the number of bullet screens, and the number of favorites; they cannot solve the problem that videos uploaded by vascular surgeons occupy only a small proportion on the first page.

One study shows that shorter interactive courses are beneficial (16). Inevitably, the attention span of adults will weaken after 15–20 min, so shorter interactive courses can promote information retention and concentration of the trainees (16). Hopkins et al. also pointed out that millennials prefer interactive shorter case discussions rather than “flipped classroom” lectures (16). Also, we found that most of the videos on Bilibili were very short (less than 10 min), and even a treatment video about a disease could be split into several small videos, which was friendly to the viewer.

Results vary with the nature of the retrieved part of speech. When searching for the name of a disease, the results were varied, including the introduction of the disease, etiology, risk factors, clinical manifestations, treatment, prognosis, and prevention. When retrieving disease treatment methods (EVAR, CAS, and varicose vein surgery) and surgical video demonstrations, there is also popular science content such as surgical indications, conservative treatment indications, and medical expense reimbursement. However, this large number of materials can be overwhelming for the students. A study indicated that after searching for femoral artery access technology on YouTube, the clear and accurate results were less than 10% (8). Our results showed that there were only 55.7% of medical-related videos, so the accuracy of retrieval needs to be improved. In addition, there were some results about Traditional Chinese Medicine, which was clearly different from other video platforms.

We believe that our results will give people an understanding of the status of videos about vascular surgery on the Bilibili platform. This study also had some limitations: first, we used 12 common search terms related to vascular surgery diseases, and a more comprehensive search is necessary for the future; second, we only analyzed the audience and uploaders of the video, and a more detailed analysis of the video content is needed.

## Conclusion

Our research suggests that although there are plentiful videos about vascular surgery on the Bilibili platform, there are few videos for professionals. In addition, there are very few vascular surgeons among video uploaders, especially in the results on the first pages, so the online presence of vascular surgeons needs to be improved.

## Data Availability

The original contributions presented in the study are included in the article/supplementary material; further inquiries can be directed to the corresponding author/s.
